# The utility of three-dimensional modeling and printing in pediatric surgical patient and family education: a systematic review

**DOI:** 10.1186/s41205-023-00198-4

**Published:** 2024-01-03

**Authors:** Angela Yang, Kapilan Panchendrabose, Cameron Leong, Syed Shuja Raza, Shahrzad Joharifard

**Affiliations:** 1 Office of Pediatric Surgical Evaluation and Innovation, British Columbia Children’s Hospital, University of British Columbia, Vancouver, BC Canada; 2 Max Rady College of Medicine, University of Manitoba, Winnipeg, MB Canada; 3 Faculty of Medicine, University of British Columbia, Vancouver, BC Canada; 4 Cumming School of Medicine, University of Calgary, Calgary, AB Canada; 5 Division of Pediatric Surgery, Department of Surgery, British Columbia Children’s Hospital, University of British Columbia, Vancouver, BC Canada

## Abstract

**Background:**

Three-dimensional (3D) modeling and printing are increasingly being used in surgical settings. This technology has several applications including pre-operative surgical planning, inter-team communication, and patient education and counseling. The majority of research on 3D technology has focused on adult populations, where it has been found to be a useful tool for educating patients across various surgical specialties. There is a dearth, however, of research on the utility of 3D modeling and printing for patient and family education in pediatric populations. Our objective was to systematically review the current literature on how this modality is being utilized in pediatric surgical settings for patient and family education and counselling.

**Methods:**

We conducted a systematic review in accordance with PRISMA and CASP guidelines. The MEDLINE, CINAHL, Embase, and Web of Science databases were searched from inception to October 21, 2023, with no restrictions on language or geographical location. Citation chaining was used to ensure relevant papers were included. Articles were doubly screened and data was extracted independently by two authors. In the case of disagreement, a third author was consulted. Any articles pertaining to 3D modeling and printing in pediatric surgical settings for patient and family education and counseling were included.

**Results:**

Six articles met inclusion criteria and were used for qualitative analysis. Two involved questionnaires given to parents of children to assess their understanding of relevant anatomy, surgical procedure, and risks after viewing conventional CT images and again after viewing a 3D-printed model. One involved a quasi-experimental study to assess young patients’ pre-operative surgical understanding and anxiety after undergoing conventional teaching as compared to after viewing a 3D storybook. One involved questionnaires given to parents of children in control and study groups to assess the usefulness of 3D printed models compared to conventional CT images in their understanding of relevant anatomy and the surgical procedure. Another study looked at the usefulness of 3D printed models compared to 2D and 3D CT images in providing caregiver understanding during the pre-operative consent process. The last article involved studying the impact of using 3D printing to help patients understand their disease and participate in decision-making processes during surgical consultations. In all six studies, utilizing 3D technology improved transfer of information between surgical team members and their patients and families.

**Conclusion:**

Our systematic review suggests that 3D modeling and printing is a useful tool for patient and family education and counselling in pediatric surgical populations. Given the very small number of published studies, further research is needed to better define the utility of this technology in pediatric settings.

## Introduction

In recent years, three-dimensional (3D) modeling and printing have been increasingly used in surgical settings. 3D modeling and printing is a process that creates virtual and physical models from computer-generated images. The process involves image acquisition using CT or MRI scans, image-processing, creation of a virtual model, and finally 3D printing into a physical model. 3D printing, more specifically, involves reproducing a patient’s specific anatomical structure into a physical object form from various materials [9]. In medical settings, fused deposition modeling (FDM), selective laser sintering (SLS), or electron beam melting (EBM), and stereolithography (SLA) are the most commonly used types [3]. Patient anatomical structures derived from CT or MRI images can also be rendered into 3D augmented reality (AR) or virtual reality (VR) spaces to demonstrate complex anatomic structures [10].

3D technology has proven to be a useful tool for pre-operative surgical planning, inter-team communication, and patient education and counseling. The majority of research on 3D technology, however, has focused on adult populations. Previous studies in adult populations have shown that 3D-printed models are useful tools during patient counseling prior to surgery [9]. Indeed, Pugliese et al. found that adult patients undergoing laparoscopic splenectomy, nephrectomy, or pancreatectomy reported a higher level of understanding of their treatment plan when a 3D model was used during consultation with the surgical team [9].

There is a relative dearth of research on the utility of 3D modeling and printing for patient and family education in pediatric surgical settings. The aim of this systematic review, therefore, is to provide a qualitative synthesis of the research currently available on the use of 3D modeling and printing in the context of patient and family education and counseling in pediatric surgical settings. In particular, this study aims to provide insight into the utility of 3D modeling and printing in improving patient and family understanding of their disease process, specific anatomy, proposed treatment plan, and, if applicable, surgical procedure. Moreover, this study investigates the utility of 3D technology in improving communication between the surgical team and the patient and their family during the consent process. This is particularly important for the pediatric setting since pediatric patients typically possess a limited understanding of human disease and express greater fear of body mutilation as compared to adults [5].

## Methods

### Data sources

We conducted a structured literature search of published articles in adherence with PRISMA guidelines for systematic reviews. The following databases were searched from inception to October 2023: MEDLINE, Evidence Based Medicine (EBM), Web of Science (WoS), Embase, and the Cumulative Index of Nursing and Allied Health Literature (CINAHL).

### Selection of articles

Since we suspected the literature on this topic would be limited, we initially searched for any English-language study pertaining to 3D modeling and printing in the context of surgical patient education. In order to capture as many articles as possible, we did not initially limit the search to pediatric patients. There were also no restrictions with respect to surgical specialty. Case reports, case reviews, and commentaries were excluded. Literature not related to the use of 3D modeling and printing in the context of surgical patient education, counseling, or consent were excluded. Literature pertaining to animals, animal models, and dental surgery and procedures were also excluded. Relevant articles from the various databases were imported into Covidence, a screening and data extraction tool.

### Search strategy

The search strategy was developed with the consultation of a University of British Columbia medical librarian. It was developed for MEDLINE and adjusted appropriately for the other databases. Search terms included: *three-dimensional printing, three-dimensional imaging, patient education, family, knowledge, teach, inform, instruct, consent.* Citation chaining was also used from the reference list of the included articles to ensure relevant papers were included.

## Results

### Literature search

**Fig. 1:**
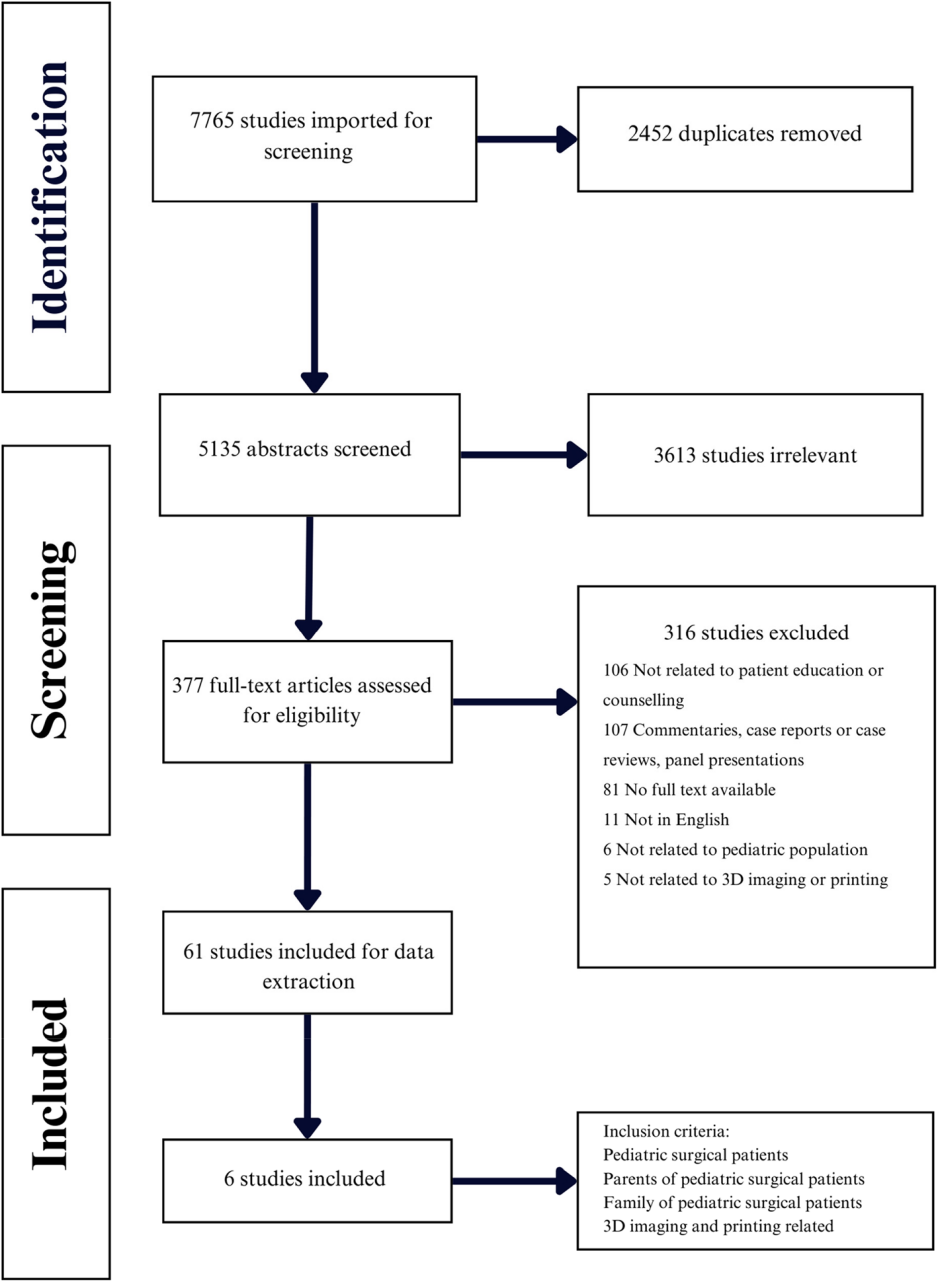
Flowchart of systematic review

Our initial search of MEDLINE, EBM, CINAHL, and WoS identified 7765 studies, of which 2452 were duplicates (Fig. 1). 5135 abstracts were screened and 377 met inclusion criteria for full-text review. After full-text review, 61 articles remained and were used for data extraction. Articles were doubly screened and in the case of disagreement, a third reviewer was consulted. After completion of data extraction, six articles met all inclusion criteria and were used for qualitative analysis.

### Included studies

Of the six articles meeting inclusion criteria, two involved questionnaires given to parents of children to assess their understanding of relevant anatomy, surgical procedure, and risks after viewing conventional CT images and again after viewing a 3D-printed model [1, 11]. One involved a quasi-experimental study to assess young patients’ pre-operative surgical understanding and anxiety after undergoing conventional teaching as compared to viewing a 3D storybook [5]. One involved questionnaires given to parents of children in control and study groups to assess the usefulness of 3D printed models compared to conventional CT images in their understanding of relevant anatomy and the surgical procedure [2]. Another study looked at the usefulness of 3D printed models compared to 2D and 3D CT images in providing caregiver understanding during the pre-operative consent process [12]. The final article involved assessing the impact of using 3D virtual model to improve patients’ understanding of their disease and participate in decision-making processes during surgical consultations. A summary of the included studies is shown in Table 1 and they are discussed in more detail below.

**Table 1:** Included studies

**First Author**	**Journal**	**Year**	**Study Type**	**Study Population**	**Surgical Subspecialty**	**Synopsis**
Alshomer, F. [1]	Journal of Craniofacial Surgery	2019	Cross-sectional study	7 pediatric patients and 14 parent participants with mean age of patients = 17.00 +/- 8.34 months.	Neurosurgery	Questionnaires were given to parents of children to assess their understanding of relevant anatomy, surgical procedure, and risks after viewing conventional CT images and again after viewing a 3D-printed model.
Deng, X. [2]	Journal of Cardiothoracic Surgery	2021	Prospective, randomized controlled study	Parents of 40 pediatric patients	Cardiothoracic Surgery	Questionnaires were given to the control group and experimental group to assess the usefulness of using a 3D printed model, compared to the use of standard 2D diagrams and verbal explanation, in improving patient understanding of relevant anatomy and proposed surgical procedure.
Konas, E. [4]	The Journal of Craniofacial Surgery	2009	Prospective study	5 patients between the ages of 9.7 and 34.6 years of age with mean age of 22.3 years.	Plastic Surgery	Assessed the impact of using 3D virtual models to improve patient’s understanding of their disease and participate in decision-making processes during surgical consultations.
Macindo, J. [5]	Association of perioperative Registered Nurses Journal	2015	Quasi-experimental study	20 children between 4-6 years of age with mean age of 5.2 +/- 0.83 years.	General, Cardiovascular, Urological, Orthopedic, ENT Surgeries	Questionnaires were administered before and after respective interventions to assess young patients’ pre-operative surgical understanding and anxiety after undergoing conventional teaching as compared to viewing a 3D storybook.
Yang, T. [11]	International Medical Research	2018	Prospective study	7 pediatric patients and 14 parent participants with mean age of patients = 30.71 +/- 24.55.	Pediatric General Surgery	Questionnaires were given to parents of children to assess their understanding of relevant anatomy, surgical procedure, and risks after viewing conventional CT images and again after viewing a 3D-printed model.
Youn, JK. [12]	Scientific Reports	2023	Prospective study	Caregivers of 8 pediatric patients under the age of 18 with median age = 4.1 (range, 1.8-18.1)	General, Oncological, Vascular Surgeries	2D CT images, 3D CT images, and 3D printed models of patient specific anatomical lesions were used to assess the usefulness of each modality for caregiver understanding during the pre-operative consent process.

Alshomer et al. conducted a cross-sectional study on the utility of patient-specific 3D-printed models in educating parents of pediatric surgical patients about craniosynostosis in Saudi Arabia. Their study included 14 parents of 7 patients (mean age of patients = 17.00 +/- 8.34 months). Importantly, the majority of parents were well educated, with 71% holding a bachelor’s degree. Researchers utilized a desktop 3D printer to create patient-specific anatomical models. They then designed and validated a Likert questionnaire to assess parental knowledge, expectations, and understanding of the proposed surgical intervention. This questionnaire was administered to parents after review of conventional CT images and then again after review of the 3D-printed model. Mean scores were compared between questionnaires. Researchers found that, after viewing the 3D model, there was a statistically-significant improvement in parental understanding of craniosynostosis and agreement with the proposed treatment plan. Interestingly, however, parents reported better understanding of potential complications after viewing conventional CT images.

Yang et al. used a similar methodology to study the impact of using 3D-printed liver modules for parental education in children with liver tumors requiring hepatectomy in China. Their study similarly included 14 parents of 7 patients (mean age of patients = 30.71 +/- 24.55 months). Parents were slightly less educated, with a mean educational level of 10 years. Researchers used MDCT images, Mimics software, and an industrial stereolithography rapid prototyping printer to segment and print their 3D models. Their true/false questionnaire aimed to quantify parental knowledge of the liver in general, their child’s specific disease, and the planned surgery. Parents completed the questionnaire after reviewing conventional CT images with the surgeon and again after reviewing the 3D model with the surgeon. Mean scores were compared. Researchers found that there was a statistically-significant improvement in parental understanding of liver anatomy, liver physiology, tumor characteristics, surgical procedure, and surgical risks.

Macindo et al. utilized a quasi-experimental approach to evaluate pre-operative knowledge and anxiety among young children undergoing major surgery in the Philippines. Twenty children aged 4-6 years (mean age 5.2 +/- 0.83 years) were randomly assigned to receive standardized traditional health teaching (*n* = 8 patients) or view a 3D storybook titled “Jared’s Hospital Adventure” (*n* = 12 patients). Children were then administered a questionnaire before and after their respective interventions and results were compared between groups. Pre-tests results assessing surgical knowledge and anxiety were similar between groups. Post-test results revealed that the 3D storybook group exhibited significantly better surgical knowledge and decreased anxiety.

Konas et al. conducted a prospective trial in Turkey to examine the utility of 3D volumetric assessments in the minimally-invasive treatment of head and neck vascular malformations using pre-operative embolization and surgical curettage. While not the main aim of the study, researchers also utilized patient-specific 3D virtual models to assess patient understanding of the anatomy of their lesions and the proposed treatment strategy. A total of 5 patients ranging in age between 9.7 and 34.6 years (mean age 22.3 years) were enrolled in the study. Researchers used high-resolution CT images and Mimics software to create virtual 3D models. The authors state that “all the patients emphasized (assessed) that the 3D demonstration of the lesions and their relation with other anatomic structures helped them to understand the extent of their pathology and aim of the proposed treatment.” However, the article does not indicate how this conclusion was reached.

Deng et al. conducted a randomized controlled study in China that looked at the usefulness of a 3D printed model of a ventricular septal defect (VSD) in the surgical consent process. They included the parents of 40 pediatric patients that were going for elective perimembranous VSD repair. A surgeon explained the disease, anatomy of the heart defect, the surgical treatment plan, and potential complications to the parents in the control group using standard 2D images taken from the Internet. The surgeon then explained the same information to the experimental group using the 3D printed VSD heart model. Afterwards, the parents were given a Linkert-type questionnaire that assessed their understanding of VSD anatomy, the procedure, potential complications, and overall understanding of the surgical consent process. The researchers noted no difference in the education level and demographics of the parents between the two groups. They found that the parents’ understanding of the VSD anatomy and surgical plan were significantly improved with the use of the 3D printed model.

Youn et al. looked at the usefulness of 3D printed models for pediatric retroperitoneal tumor resection on educating medical students, residents, pediatric surgeons, and informing parents/legal guardians of pediatric patients during the pre-operative consent process. The researchers used three types of modules during the explanatory process: 2D CT images, 3D reconstructed CT images, and 3D printed models. Information about the disease was presented to the patients’ caregivers before the surgery using the three modules in succession and their understanding was assessed. However, it is unclear from the article how this was done. The results showed that the use of the 3D printed model was most useful for the patients’ caregivers in understanding the lesion.

### Thematic analysis

The main theme that emerged from our analysis was improved patient or parental knowledge of anatomy and the proposed surgical procedure after being exposed to 3D technology, be that virtual 3D models, printed 3D models, or a 3D storybook.

3D-printed anatomical models can be used to provide information about the specifics of the patient’s medical condition to pediatric patients and their family members. In their cross-sectional study investigating the use of 3D-printed craniofacial models and 3D models of CT images, Alshomer et al. found that the parents were more agreeable to the surgical plan when the 3D-printed patient-specific model was used [1]. Parental understanding of potential surgical complications also improved when 3D models of CT images were used during consultations [1]. In Deng et al.’s study, the parents gave a high overall rating for the usefulness of a 3D model in providing a understanding of VSD anatomy and the surgical plan. Similarly, Yang et al. found that the use of 3D printing improved parental understanding of basic liver anatomy and physiology as well as understanding of the surgical plan and associated risks [11]. Youn et al. found that using the 3D printed models in addition to 2D CT images improved the guardians’ understanding and satisfaction of the surgical treatment plan, which aligns with the findings from Yang et al.’s study [12]. Although their methodology is less clear, Konas et al. also found that using 3D modeling in the treatment of head and neck soft tissue vascular lesions facilitated patients’ ability to visualize the lesion in relation to other anatomical structures, which, in turn, helped patients better understand the treatment plan [4]. Finally, in their quasi-experimental study using a 3D storybook to assess surgical knowledge and pre-operative anxiety levels among young children, Macindo et al. found that pediatric patients in the experimental group demonstrated improved surgical knowledge and decreased anxiety after viewing a 3D storybook as compared to after receiving standard teaching [5].

## Discussion

This is the first study to provide an overview of the current research on the use of 3D modeling and printing for patient education and counselling in pediatric surgical settings. 3D models, be their virtual or printed, were found to be useful tools during pediatric surgical consultations. All included studies found that using 3D technology improved the knowledge and understanding of patients and their families regarding patient-specific anatomy and treatment plans. Improving baseline understanding enhances patient and parental autonomy and invites the patient and family to participate more fully in a shared decision-making process with the surgeon [7].

Treating pediatric patients involves working with their parents and other family members. Often, parental understanding can be representative of their child’s understanding [11]. Further, involving pediatric patients in the surgical consultation can be challenging due to age, level of maturity, shorter attention spans, limited vocabularies, fear of body mutilation, and limited ability to comprehend complex medical conditions [5]. In addition, pediatric patients often experience significant anxiety, which is frequently exacerbated by lack of age-appropriate pre-operative counselling. Despite these challenges, involving children in the consultation process is of great importance. Using 3D technology, such as virtual 3D models, printed 3D models, or 3D storybooks can help mitigate these negative feelings and instill greater trust and confidence in the surgical team and the proposed treatment plan [6].

Utilizing 3D technology can also help improve communication between the surgeon and patient and their family, which can help reduce anxiety and stress that is usually associated with undergoing surgery [5]. Surgeons often provide patient and family education throughout the treatment process using CT or MRI images to convey disease information, explain the proposed procedure, and explain risks and benefits of proceeding with surgery. It may be challenging for individuals without a medical background, especially those with a limited education, to fully understand this information. Virtual and physical 3D models can allow patients and family to better visualize what is being described to them. Further, patients and their family members may feel overwhelmed during the surgical consultation, so being able to see their CT or MRI imaging in a virtual or physical 3D model may help mitigate feelings of stress and anxiety [7]. There is further potential for this technology to be modified for use in an age-appropriate manner for pediatric patients, just as Macindo’s team did with their 3D storybook.

A strength of our study is that we conducted very broad search of multiple databases and included a large number of studies in our initial screening. Specifically, we did not limit our initial search to pediatric studies, which allowed us to identify Konas et al.’s study, which included a mixed pediatric and adult population. However, our review is limited due to the small number of relevant articles identified. By restricting our search to exclude case studies, commentaries, and review articles, the number of included studies was significantly reduced. Therefore, it is difficult to draw any conclusions with certainty given that only four studies were ultimately included.

Due the qualitative nature of our study, heterogeneity of surgical procedures included, and different methodologies used to measure patient understanding, it is difficult to compare results between studies. On the other hand, one obvious limitation of the questionnaire methodology utilized by Alshomer et al. and Yang et al. is that improved understanding may have resulted from having been exposed to the information twice—once using CT images and again using the 3D model—and not from using the 3D model. Another significant limitation of Konas et al.’s study methodology is that they do not stipulate how they assessed patient understanding. In addition, there is the possibility of response biases in several of the included studies. One type of response bias of particular concern is demand characteristics, which occurs when participants alter their behavior, such as their responses in follow-up questionnaires, simply because they know they are part of a research study [8].

## Conclusion

Providing pre-operative counselling to pediatric patients and their families is a unique and challenging process. In contrast to adult populations, preparing pediatric populations for surgery must include their parents, caregivers, and other family members. Although there is a growing body of literature around the use of 3D modeling and printing in surgical settings, most of this research focuses on adult populations. The literature investigating the use of 3D modeling and printing in the pediatric literature is sparse and often consists of case reports and case series. The available literature does, however, suggest that 3D technology is a useful adjunct in improving communication between patients and their families and the surgical team. Using this technology, furthermore, can improve understanding and knowledge of patient-specific anatomy and the proposed surgical procedure. Additional research is needed to assess the utility of 3D modeling and printing for patient education and counselling in pediatric populations. These studies should aim to include larger sample sizes and attempt to control for response bias.

## Data Availability

Not applicable.
